# Five Years’ Experience with Gene Panel Sequencing in Hereditary Hemolytic Anemia Screened by Routine Peripheral Blood Smear Examination

**DOI:** 10.3390/diagnostics13040770

**Published:** 2023-02-17

**Authors:** Namsu Kim, Tae Yun Kim, Ji Yoon Han, Joonhong Park

**Affiliations:** 1 Department of Laboratory Medicine, Jeonbuk National University Medical School and Hospital, Jeonju 54907, Republic of Korea; 2 Department of Thoracic and Cardiovascular Surgery, Jeonbuk National University Medical School and Hospital, Jeonju 54907, Republic of Korea; 3 Department of Pediatrics, College of Medicine, The Catholic University of Korea, Seoul 06591, Republic of Korea; 4 Research Institute of Clinical Medicine, Jeonbuk National University-Biomedical Research Institute, Jeonbuk National University Hospital, Jeonju 54907, Republic of Korea

**Keywords:** gene panel sequencing, hereditary hemolytic anemia, *ANK1* gene, *EPB41* gene, *SPTB* gene, *HBB* gene, Ion Torrent PGM™ Dx System

## Abstract

Background: Hereditary hemolytic anemia (HHA) is defined as a group of heterogeneous and rare diseases caused by defects of red blood cell (RBC) metabolism and RBC membrane, which leads to lysis or premature clearance. The aim of this study was to investigate individuals with HHA for potential disease-causing variants in 33 genes reported to be associated with HHA. Methods: A total of 14 independent individuals or families diagnosed with suspected HHA, and in particular, RBC membranopathy, RBC enzymopathy, and hemoglobinopathy, were collected after routine peripheral blood smear testing. A custom designed panel, including the 33 genes, was performed using gene panel sequencing on the Ion Torrent PGM™ Dx System. The best candidate disease-causing variants were confirmed by Sanger sequencing. Results: Several variants of the HHA-associated genes were detected in 10 out of 14 suspected HHA individuals. After excluding those variants predicted to be benign, 10 pathogenic variants and 1 variant of uncertain significance (VUS) were confirmed in 10 individuals with suspected HHA. Of these variants, the p.Trp704Ter nonsense variant of *EPB41* and missense p.Gly151Asp variant of *SPTA1* were identified in two out of four hereditary elliptocytoses. The frameshift p.Leu884GlyfsTer27 variant of *ANK1*, nonsense p.Trp652Ter variant of the *SPTB*, and missense p.Arg490Trp variant of *PKLR* were detected in all four hereditary spherocytosis cases. Missense p.Glu27Lys, nonsense p.Lys18Ter variants, and splicing errors such as c.92 + 1G > T and c.315 + 1G > A within *HBB* were identified in four beta thalassemia cases. Conclusions: This study provides a snapshot of the genetic alterations in a cohort of Korean HHA individuals and demonstrates the clinical utility of using gene panels in HHA. Genetic results can provide precise clinical diagnosis and guidance regarding medical treatment and management for some individuals.

## 1. Introduction

Hereditary hemolytic anemia (HHA) is defined as a group of heterogeneous and rare diseases caused by defects of red blood cell (RBC) metabolism and/or defects of the plasma membrane, which leads to the lysis or premature clearance of these cells [1]. Representative laboratory findings comprise decreased haptoglobin, increased lactate dehydrogenase (LDH) and unconjugated bilirubin, reticulocytosis, abnormal RBC morphology, and often iron overload. The clinical manifestations include hemolytic anemia of variable degree, gallstones, jaundice, and splenomegaly. HHA is diagnosed according to persistent hemolytic anemia, laboratory testing, clinical evaluation, and family history. Overlaps in hematological and clinical characteristics exist among the different forms of HHAs, making differential diagnosis difficult, in particular for atypical and mild types. HHA has classically been categorized into three distinct types: RBC membranopathy [2], RBC enzymopathy [3], and hemoglobinopathy [4]. In the epidemiological study of hereditary hemolytic anemia in the Korean pediatric population, the incidence of newly diagnosed pediatric patients with HHA in the Republic of Korea from 2007 to 2016 was compared to that of an HHA cohort from 1997 to 2006 [5]. As a result, RBC membranopathies were observed to be the most common form of HHA (in an earlier period from 1997 to 2006, the incidence was 87.1%, compared to a later period from 2007 to 2016, in which it was 71.3%). When multi-gene panel sequencing was applied to these RBC membranopathies, the most frequent genetic variants in HS were found in *SPTB*, followed by *ANK1* and *SLC4A1* [6]. Significant increases in RBC enzymopathies (in the earlier period: 2.1%; in the later period: 6.2%) and hemoglobinopathies (in the earlier period: 4.2%; in the later period: 16.0%) were also found over that time. Korean individuals with RBC enzymopathy have distinct hemolytic symptoms such as relatively low Hb and haptoglobin levels but high LDH levels at very young ages.

On the other hand, none of the screening tests when used alone can reach a definitive diagnosis in 100% of cases, and some individuals remain undiagnosed even after extensive and complete investigations. For example, the eosin-5-maleimide (EMA) binding test and ektacytometry are tools used for the screening of membrane disorders, and when combined, they have the best performance in detecting HS [7]. New genetic approaches are currently being developed to automate the diagnostic yield and to expand molecular characterization. Genetic alterations may contribute to a considerable proportion of HHA individuals. At least 70 genes have been reported to be disease-associated. As such, a fast, effective genetic screening approach for HHA cases would be useful [8]. Recently, molecular testing advances have permitted definitive diagnoses in certain situations, and its use is increasing thanks to the development of next-generation sequencing (NGS) techniques [9]. The gene panel sequencing of multiple genes of interest is becoming more frequently applied in medical laboratories and is a rapid, cost-effective alternative to whole-genome sequencing or whole-exome sequencing. Benchtop sequencers have substantial time and cost savings over conventional Sanger sequencing and have the advantages of easy-to-interpret results, flexible sequencing options, and low cost compared to high-throughput sequencers [10]. The diagnostic success rate of NGS is higher when used in combination with conventional methods, together with a clinical classification and detailed symptoms of the individuals [11]. With a precise genetic diagnosis, physicians can provide genetic counseling and accurate treatment strategies for the management of HHA individuals and their family members. However, functional tests are often needed to confirm the pathogenicity of the detected variants [7].

The aim of this study was to identify pathogenic variants in 33 candidate genes associated with HHA in a cohort of individuals with various types of HHA and to illustrate the implementation of variant detection using gene panel sequencing after routine PB smear testing.

## 2. Materials and Methods

### 2.1. Specimen Collection and DNA Extraction

**Figure 1:**
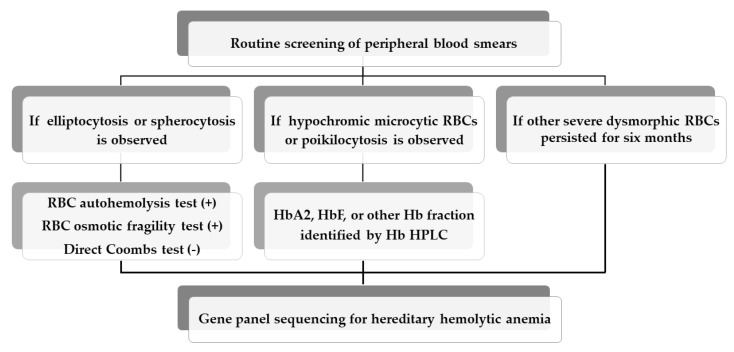
A test workflow for the screening and molecular diagnosis of suspected hereditary hemolytic anemia. (+), positive for test; (-), negative for test.

The annual number of routine PB smear examinations requested at the Department of Laboratory Medicine, Daejeon St. Mary’s Hospital (Daejeon, Republic of Korea) were 3534 in 2015, 4437 in 2016, 4677 in 2017, 4446 in 2018, and 4694 in 2019, respectively. If more than 50% abnormal or dysmorphic RBCs were observed upon the routine screening of peripheral blood smears, additional laboratory testing was performed for differential diagnosis. Briefly, an RBC autohemolysis test, RBC osmotic fragility test, and direct Coombs test were conducted to determine if hereditary elliptocytosis (HE), hereditary spherocytosis (HS), or hereditary stomatocytosis (HSt) was suspected. The high-performance liquid chromatography (HPLC) of hemoglobin (Hb) using an HLC-723G8 HPLC analyzer (Tosoh Co., Tokyo, Japan) was performed to characterize abnormal Hb variants such as *HBA2*, HbF, or other Hb fractions found commonly in beta thalassemia major (BTM) and beta thalassemia minor (BTm). A workflow for the screening and molecular diagnosis of suspected HHA is illustrated in Figure 1. Peripheral whole blood was collected from individuals with suspected HHA and their family members if available. Genomic DNA was extracted from peripheral blood leukocytes using a QIAamp DNA Blood Mini Kit (Qiagen, Hilden, Germany) according to the manufacturer’s protocols.

### 2.2. Panel Design and Library Preparation

Thirty-three candidate genes have been reported to be associated with HHA and were selected for panel design based on PubMed literature retrieval (https://pubmed.ncbi.nlm.nih.gov/, accessed on 18 March 2020) and Online Mendelian Inheritance in Man search (OMIM, https://omim.org/, accessed on 18 March 2020). The 33 selected genes in a custom HHA panel are summarized in Table 1. Primers of overlapping amplicons covering the coding sequences of exons and exon–intron boundaries of each selected gene were designed automatically by Ion AmpliSeq Designer (https://www.ampliseq.com/, accessed on 30 March 2020). This produced 1063 amplicons, which were divided into two Ion AmpliSeq On-Demand Panel primer pools (Life Technologies; Thermo Fisher Scientific, Carlsbad, CA, USA). Amplicon libraries were prepared using Ion AmpliSeq On-Demand Panel primer pools and the Ion AmpliSeq Library Kit Plus (Life Technologies; Thermo Fisher Scientific) and custom designed primer pools, according to the manufacturer’s protocols. The libraries prepared for the custom HHA panel were partially digested, phosphorylated using the FuPa reagent, and then ligated with barcoded sequencing adaptors using the Ion Xpress Barcode Adapter 17–32 Kit (Life Technologies; Thermo Fisher Scientific). The library was purified and quantified with a Qubit 2.0 fluorometer (Invitrogen; Thermo Fisher Scientific, Waltham, MA, USA).

### 2.3. Gene Panel Sequencing and Bioinformatics Analysis

Gene panel sequencing using an Ion Torrent PGM™ Dx System (Life Technologies; Thermo Fisher Scientific) was carried out in individuals showing abnormal RBC morphology and positive for additional laboratory testing. Briefly, seven barcoded, multiplexed samples per chip were pooled in equimolar volumes at a concentration of 50 pM and loaded onto an Ion 318 v2 chip using the Ion Chef with the Ion 318 Chip Kit (Life Technologies; Thermo Fisher Scientific). Enriched template-positive ISPs were sequenced on an Ion 318 v2 chip using Ion Torrent PGM™ Dx System with the Ion PGM HI-Q SEQ Kit (Life Technologies; Thermo Fisher Scientific) according to the manufacturer’s guidelines. Data from Ion Torrent PGM™ Dx System runs were processed automatically using Torrent Suite 5.10 and Ion Reporter 5.10 (https://ionreporter.thermofisher.com/ir/, accessed on 16 June 2020) to generate sequence reads. Genome Reference Consortium Human Build 37 (GRCh37/hg19) was used as the reference sequence for mapping alignment. After sequence alignment and variant calling, variant allelic frequency (VAF) ≥ 0.01 in the gnomAD database (https://gnomad.broadinstitute.org/, accessed on 23 June 2020), intronic variants far away from the exon–intron boundaries, and synonymous variants predicted to cause no splicing error, estimated by SpliceAI (https://spliceailookup.broadinstitute.org/, accessed on 23 June 2020), were excluded from further interpretation. Sequence reads of the candidate genes were visualized using an integrated genomics viewer (IGV). Bidirectional Sanger sequencing of the polymerase chain reaction (PCR) products was performed to confirm candidate sequence variants using the BigDye Terminator v3.1 Cycle Sequencing Kit (Applied Biosystems, Foster City, CA, USA) and was resolved by capillary electrophoresis on a 3500xL Dx Genetic Analyzer (Applied Biosystems, Carlsbad, CA, USA). VarSome was used as an impact analysis tool, aggregator, and search engine for human genetic variation (https://varsome.com/, accessed on 30 June 2020). We placed verified variants into the following categories: benign (B), likely benign (LB), likely pathogenic (LP), pathogenic (P), and variant of uncertain significance (VUS), based on guidelines from the American College of Medical Genetics and Genomics (ACMG) and Association of Molecular Pathology (AMP) [12].

## 3. Results

### 3.1. Sequencing Quality Metrics and Overall Custom Designed Performance

In sequencing quality metrics for raw DNA sequencing data generated by the Ion Torrent PGM™ Dx System (Life Technologies; Thermo Fisher Scientific), 691 fragments with a target size of about 204.8 kb were produced simultaneously from about 1063 amplicons targeted on the exon–intron boundaries and exons of 33 HHA-associated genes. The custom-designed gene panel covered 94.2% of the bases in the target sites. Amplicon sizes, with an average amplicon size of 157 bp, ranged from 93 to 227 bp. For each specimen, coverage of the targeted site was about 97.1%, with a mean read depth of 164×. Target coverage read depth at 30× was >95%, with a mean read size of 177 bp. After initial variant calling by Torrent Suite 5.10 and Ion Reporter 5.10, more than 50 variants were identified in each specimen. After variants were filtered by variant type, VAF, and inheritance pattern, only one or two candidate variants from each specimen required confirmation by Sanger sequencing.

### 3.2. Molecular Diagnosis of the Suspected Hereditary Hemolytic Anemia

**Figure 2:**
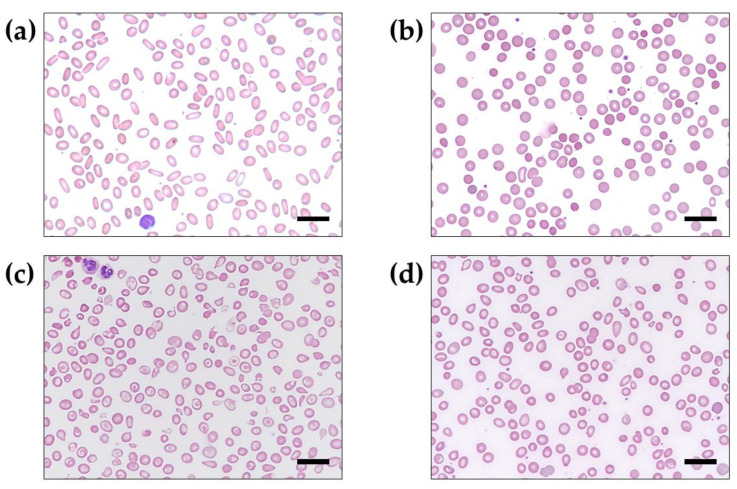
Results of peripheral blood smear examination in individuals with suspected hereditary hemolytic anemia (Wright–Giemsa stained; 1000× magnification). (**a**) Elliptocytes appeared oval or elongated, from slightly egg-shaped to rod or pencil forms in an individual with suspected hereditary elliptocytosis (D099). (**b**) Spherocytes appeared smaller and darker than normocytic red blood cells and lacked an area of central pallor in an individual with suspected hereditary spherocytosis (D131). (**c**) Severe hypochromic microcytic RBCs and poikilocytosis in a Cambodian individual with suspected beta thalassemia major (D136). (**d**) Mild to moderate hypochromic microcytic RBCs and poikilocytosis in a Chinese individual with suspected beta thalassemia minor (D888). Scale bar = 20 μm.

A total of 21,788 routine PB smear tests were examined between January 2015 and January 2019. Among them, 14 individuals (0.064%, 14/21,788) were suspected of HHA based on the results of abnormal RBC morphology as well as being positive upon additional laboratory testing. There were four HE, four HS, five BT, and one Hst. For example, PB smears revealed distinctly abnormal RBC morphologies in each individual with suspected HE (D099 in Figure 2a), HS (D131 in Figure 2b), BTM (D136 in Figure 2c), and BTm (D888 in Figure 2d).

Gene panel sequencing was performed to identify molecular defects responsible for the suspected HHA individuals. From this analysis, several variants of the different HHA-associated genes were detected in 10 out of 14 suspected HHA individuals. In two out of four HE, a nonsense variant in *EPB41* (reference transcript ID: NM_001166005.2:c.2112G > A/p.Trp704Ter) [13] and a missense variant in *SPTA1* NM_003126.4:c.452G > A/p.Gly151Asp) were identified. In all four HS, a frameshift variant in *ANK1* (NM_001142446.2:c.2642_2645dup/p.Leu884GlyfsTer27), a nonsense variant in *SPTB* (NM_001024858.3:c.1956G > A/p.Trp652Ter) [14], and a missense variant in *PKLR* (NM_000298.6:c.1468C > T/p.Arg490Trp) were detected. In four out of five BT, including one BTM and four BTm, missense (NM_000518.5:c.79G > A/p.Glu27Lys), splicing error (c.92 + 1G > T and c.315 + 1G > A), and nonsense (c.52A > T/p.Lys18Ter) variants in *HBB* were identified. No variant was detected in an individual with suspected HSt. All variant annotation information confirmed in this study is summarized in Table 2.

## 4. Discussion

The analysis of RBC morphology from PB smears is often the first indicator of the HHA under investigation and is still a fundamental test in the diagnostic algorithm. The detection of elliptocytes, spherocytes, or stomatocytes, although they may be present in differing and sometimes low numbers, addresses the extension of the diagnostic workflow toward an RBC membranopathy. In the situation of an RBC enzymopathy, the RBC morphology is usually reported to not be specific, but some abnormalities, although unremarkable, may still be suggestive of a particular deficiency such as prominent basophilic stippling in pyrimidine-5ʹ-nucleotidase deficiency or the presence of echinocytes in triose phosphate isomerase or PK deficiency [15]. However, the manual analysis of RBC morphology is hampered by a lack of standardization, is time-consuming, and is prone to some degree of subjectivity [16]. Cell shape classification and identification using artificial intelligence algorithms based on artificial neural networks may lead to a more rapid, effective, and standardized analysis of RBC morphology, but the challenge with these automated analyses is that artificial neural networks need to be customized, trained, and validated [17].

The clinical and genetic diagnosis of individuals with HHA may be extremely difficult to obtain. For some situations, the high symptom variability can be partially explained either by reduced penetrance or by high genetic heterogeneity and variable expressivity [18]. Significant progress has been made in discovering new disease-associated genes involved in red blood cell disorders, and increasing genetic heterogeneity underscores the difficulty of performing a very complex differential diagnosis [19]. It is appropriate in this context to consider gene panel sequencing. Several studies analyzed cohorts of patients ranging from 19 to 57 individuals using gene panel sequencing comprising a smaller gene set of 28 to 40 loci causative of some RBC membranopathies, RBC enzymopathies, sideroblastic anemia, Diamond–Blackfan anemia, and congenital dyserythropoietic anemia [20,21]. Russo and colleagues reported a diagnostic workflow for HHA, based on the development of two comprehensive gene sets, including 34 and 71 genes, respectively. As a result, they demonstrated that the multi-gene approach is valuable for guiding treatment as well as for achieving accurate and definitive diagnosis [8].

In this study, we demonstrate the feasibility of implementing broad-based, flexible, NGS-based gene panel sequencing to meet the clinical molecular testing need after routine PB smear testing. At the same time as reviewing patient medical records, three criteria are applied to ensure the successful implementation of this test workflow. First, if elliptocytosis or spherocytosis is observed by routine PB smear testing, additional testing including the RBC autohemolysis test, RBC osmotic fragility test, and Direct Coombs test are performed to screen for presumptive HE or HS. Second, if hypochromic microcytic RBCs or poikilocytosis are observed, Hb HPLC is performed to identify *HBA2*, HbF, or other Hb fractions, which are frequently observed in alpha or beta thalassemia. Third, if other severe dysmorphic RBCs persist for six months, gene panel sequencing for HHA is directly performed for genetic diagnosis. In our study, 14 individuals with suspected HHAs, including 9 RBC membranopathy and 5 hemoglobinopathy cases, were selected for gene panel sequencing. From this, several variants of the different HHA-associated genes were detected in 10 out of 14 suspected HHA individuals. After excluding variants predicted to be benign, 10 pathogenic variants and 1 variant of uncertain significance (VUS) were confirmed in 10 individuals with suspected HHA. Of these, the nonsense variant (p.Trp704Ter) in *EPB41* and missense variant (p.Gly151Asp) in *SPTA1* were identified in two out of four HEs. A frameshift variant (p.Leu884GlyfsTer27) in *ANK1*, nonsense variant (p.Trp652Ter) in *SPTB*, and missense variant (p.Arg490Trp) in *PKLR* were detected in all four HSs. Missense (p.Glu27Lys) and nonsense (p.Lys18Ter) variants and splicing errors such as c.92 + 1G > T and c.315 + 1G > A of the *HBB* were identified in four out of five BTs.

The most common RBC membranopathy is HS, followed by relatively uncommon conditions, such as HE and hereditary pyropoikilocytosis. Deleterious variants in genes *ANK1*, *EPB41*, *EPB42*, *SLC4A1*, *SLC4A1*, and *SPTB* are known to cause RBC membrane defects. In our study, nine individuals (four HEs, four HSs, and one Hst) were suspected of having an RBC membranopathy, and one HE and four HSs were observed to carry a pathogenic variant in one of the genes, having arrived at the diagnosis of RBC membranopathy on the basis of the conventional RBC autohemolysis test, RBC osmotic fragility test, and Direct Coombs test. The pathogenic nonsense variant p.Trp704Ter in *EPB41* was found in one HE individual, and VUS in *SPTA1* was detected in one HE individual. Although this p.Gly151Asp variant in *SPTA1* was predicted to be pathogenic by in silico analysis (SIFT 0.001; PROVEAN -4.67; Mutation assessor 3.385), a functional study is required to establish causality for the clinical manifestation. RBC enzymopathies generally present with normal morphologies of RBCs, with normocytic normochromic hemolytic anemia in the PB [22]. Thus, if an individual shows non-immune hemolytic anemia without the clinical features of hemoglobinopathy or RBC membranopathy, an RBC enzymopathy can be clinically suspected. The diagnosis of an RBC enzymopathy is based on the genetic characterization of the defect at the DNA level and reduced specific enzyme activity [22]. Among RBC enzymopathies, G6PD deficiency, the most common red cell enzyme deficiency worldwide, may be missed in severe individuals that have chronic hemolysis with markedly high reticulocytosis or require blood transfusions. Unfortunately, RBC enzymopathies such as G6PD deficiency or PK deficiency were diagnosed, based on RBC enzyme level and external single gene testing, before our gene panel sequencing was applied during the study period. RBC enzyme evaluations for glucose-6-phosphate dehydrogenase (G6PD) and pyruvate kinase (PK) activity were referred to an external medical laboratory, GC Labs (Yongin, Republic of Korea), when a disease condition was suspected by a clinician. Usually, RBC enzymopathy cannot be diagnosed by PB smear testing alone, because RBC morphology is not specific in individuals with G6PD or PK deficiencies. Even though an RBC enzymopathy was not genetically diagnosed, a missense p.Arg490Trp variant of the *PKLR* was identified in clinically suspected HS (D034) [23,24]. Interestingly, concomitant HS and PK deficiencies were reported in a Spanish family with chronic hemolytic anemia [25]. Both a heterozygous missense p.Arg216Gln variant in *SPTB* and a heterozygous missense p.Arg569Gln variant of the *PKLR* were identified in the family member. After 6 years of clinical follow-up of the patients with HS, it was inferred that the chronic hemolytic anemia may be attributable to the *SPTB* mutation only, and not the influence of the concomitant *PKLR*. Moreover, only the family members with the *SPTB* mutation exhibited an ektacytometric profile characteristic of HS. To date, the co-existence of PKD and HS is very rare, and only a few cases have been reported [26,27,28]. However, heterozygous PKD may not modulate the clinical expression of HS.

In studies of hemoglobinopathy in the Korean population, the incidence of thalassemia in young Koreans has been shown to be increasing because of increasing rates of immigration or the increase in interracial marriages between Korean males and South East Asian females in recent years [29,30]. In particular, thalassemia is highly prevalent in Southeast Asia but rare in the Republic of Korea. However, with increasing Southeast Asian immigration, cases are increasing [29]. In a prospective, observational, multicenter study [31], eighteen multiethnic subjects and four Korean subjects were tested for α-globin and β-globin gene mutations. Within the multiethnic group, five subjects (1.5%) were α-thalassemia carriers, and six (1.9%) were β-thalassemia minor. The SEA deletion in *HBA1* and *HBA2*, and the c. 126_129delCTTT (p.Phe42Leufs*19) mutation of *HBB* were the dominant inherited mutations. In our study, we had five BT individuals; one BTM with compound p.Glu27Lys and c.92 + 1G > T variants of the *HBB* was Cambodian, and the other BTm with heterozygous p.Lys18Ter *HBB* variant was Chinese. The last three Btms were Korean. Since the majority of Korean patients with hemoglobinopathy have BTm, hemolysis among these patients was not clinically significant [5]. On the other hand, hemoglobinopathy results in markedly aberrant Hb electrophoresis. Therefore, several Korean β-thalassemia patients were diagnosed based on family history and Hb electrophoresis, without genetic testing. However, since *HBB* gene sequencing is not very expensive in Korea, genetic tests were often performed when medical staff suspected β-thalassemia. By way of contrast, in the case of α-thalassemia, Hb electrophoresis test results are normal, and MLPA of *HBA1*/*HBA2* can diagnose the most common deletions. In Korea, however, MLPA is not covered by the National Health Insurance System and is therefore very expensive. Thus, in Korea, the diagnosis of α-thalassemia is considered to be underestimated [5].

There are some inherent limitations to this study. (1) The studied sample size was quite small, even though several patients were genetically confirmed as HE, HS, or BT. Further studies with large sample sizes are required. (2) Only samples showing RBC morphology abnormalities screened by PB smear were genetically analyzed. Particularly, the possibility of RBC enzymopathy cannot be ruled out, because RBC morphology is not specific in RBC enzymopathy. (3) Only 33 genes related to IHA were estimated. It is not possible to exclude other IHA-associated gene abnormalities that are not included. Future molecular studies with larger multi-gene panel are needed for the comprehensive understanding of genetic diagnosis for IHA.

## 5. Conclusions

In summary, we designed an Ampliseq-based custom panel for the genetic screening of HHA individuals and detected 10 pathogenic variants and 1 VUS. Although a relatively limited number of individuals were selected in our cohort, the results provide an overview of molecular characteristics present in HHA. Mutation-negative individuals from this cohort will require additional tests to discover the potential molecular defects causing their HHA, although we cannot exclude the concept that genetic factors are not main causes of disease in these individuals.

## Figures and Tables

**Table 1:** A list of 33 selected genes in a custom hereditary hemolytic anemia panel.

Gene	Location	Disease Phenotypes	# OMIM	Inheritance
RBC membranopathy (N = 10)				
*EPB41*	1p35.3	Elliptocytosis-1	# 611804	AD, AR
*SPTA1*	1q23.1	Elliptocytosis-2 Pyropoikilocytosis Spherocytosis-3	# 130600 # 266140 # 270970	AD AR AR
*SPTB*	14q23.3	Elliptocytosis-3 Spherocytosis-2	# 617948 # 616649	AD
*ANK1*	8p11.21	Spherocytosis-1	# 182900	AD, AR
*SLC4A1*	17q21.31	Spherocytosis-4	# 612653	AD
*EPB42*	15q15.2	Spherocytosis-5	# 612690	AD
*PIEZO1*	16q24.3	Dehydrated hereditary stomatocytosis	# 194380	AD
*KCNN4*	19q13.31	Dehydrated hereditary stomatocytosis-2	# 616689	AD
*RHAG*	6p12.3	Overhydrated hereditary stomatocytosis	# 185000	AD
*SEC23B*	20p11.23	Dyserythropoietic anemia, congenital, 2	# 224100	AR
RBC enzymopathy (N = 17)				
*ABCB7*	Xq13.3	Anemia, sideroblastic, with ataxia	# 301310	XLR
*AK1*	9q34.11	Hemolytic anemia due to adenylate kinase deficiency	# 612631	AR
*ALAS2*	Xp11.21	Anemia, sideroblastic, 1 Protoporphyria, erythropoietic, X-linked	# 300751 # 300752	XLR XL
*ALDOA*	16p11.2	Glycogen storage disease XII	# 611881	AR
*CYB5R3*	22q13.2	Methemoglobinemia	# 250800	AR
*G6PD*	Xq28	Hemolytic anemia, G6PD deficient	# 300908	XLD
*GCLC*	6p12.1	Hemolytic anemia due to gamma-glutamylcysteine synthetase deficiency	# 230450	AR
*GPI*	19q13.11	Hemolytic anemia, nonspherocytic, due to glucose phosphate isomerase deficiency	# 613470	AR
*GSS*	20q11.22	Hemolytic anemia due to glutathione synthetase deficiency	# 231900	AR
*HK1*	10q22.1	Hemolytic anemia due to hexokinase deficiency	# 235700	AR
*NT5C3A*	7p14.3	Anemia, hemolytic, due to UMPH1 deficiency	# 266120	AR
*PFKM*	12q13.11	Glycogen storage disease VII	# 232800	AR
*PGK1*	Xq21.1	Phosphoglycerate kinase 1 deficiency	# 300653	XLR
*PKLR*	1q22	Pyruvate kinase deficiency	# 266200	AR
*SLC25A38*	3p22.1	Anemia, sideroblastic, 2, pyridoxine-refractory	# 205950	AR
*TPI1*	12p13.31	Hemolytic anemia due to triosephosphate isomerase deficiency	# 615512	AR
*UGT1A1*	2q37.1	Crigler–Najjar syndrome, type I Crigler–Najjar syndrome, type II Hyperbilirubinemia, familial transient neonatal	# 218800 # 606785 # 237900	AR AR AR
Hemoglobinopathy (N = 6)				
*HBA1*	16p13.3	Thalassemia, alpha	# 604131	AR
*HBA2*	16p13.3	Thalassemia, alpha	# 604131	AR
*HBB*	11p15.4	Thalassemia, beta Delta-beta thalassemia Sickle cell anemia	# 613985 # 141749 # 603903	AD, AR AD AR
*HBD*	11p15.4	Thalassemia, delta	na	AR
*HBG1*	11p15.4	Fetal hemoglobin quantitative trait locus 1	# 141749	AD
*HBG2*	11p15.4	Fetal hemoglobin quantitative trait locus 1	# 141749	AD

RBC, red blood cell; OMIM, Online Mendelian Inheritance in Man; AD, autosomal dominant; AR, autosomal recessive; XLR, X-linked recessive; XLD, X-linked dominant; na, not available.

**Table 2:** Candidate causative variants identified in 10 individuals with hereditary hemolytic anemia.

Case ID	S/A	Dx	Gene	Transcript	Base Change	AA Change	Variant Type	Zygosity	Effect	gnomAD
D099	F/89	HE	*EPB41*	NM_001166005.2	c.2112G > A	p.Trp704Ter	Nonsense	Het	P	nf
D050	M/89	HE	*SPTA1*	NM_003126.4	c.452G > A	p.Gly151Asp	Missense	Het	VUS	0.000158
D131	M/62	HS	*ANK1*	NM_001142446.2	c.2642_2645dup	p.Leu884GlyfsTer27	Frameshift	Het	P	nf
D112 *	F/70	HS	*SPTB*	NM_001024858.3	c.1956G > A	p.Trp652Ter	Nonsense	Het	P	nf
D114 *	M/48	HS	*SPTB*	NM_001024858.3	c.1956G > A	p.Trp652Ter	Nonsense	Het	P	nf
D034	F/89	HS	*PKLR*	NM_000298.6	c.1468C > T	p.Arg490Trp	Missense	Het	P	0.0000637
D136	M/27	BTM	*HBB*	NM_000518.5	c.79G > A	p.Glu27Lys	Missense	Het	P	0.0000637
			*HBB*	NM_000518.5	c.92 + 1G > T		Splicing error	Het	P	nf
D129	M/76	BTm	*HBB*	NM_000518.5	c.315 + 1G > A		Splicing error	Het	P	0.0000319
D009	F/81	BTm	*HBB*	NM_000518.5	c.315 + 1G > A		Splicing error	Het	P	0.0000319
D888	F/41	BTm	*HBB*	NM_000518.5	c.52A > T	p.Lys18Ter	Nonsense	Het	P	0.0000637

* They are biological mother and son relationships. S/A, sex/age; Dx, provisional diagnosis at the time of admission; HE, hereditary elliptocytosis; HS, hereditary spherocytosis; BTM, beta thalassemia major; BTm, beta thalassemia, minor; AA, amino acid; Het, heterozygous, P, pathogenic; VUS, variant of uncertain significance; gnomAD, frequencies in whole genome sequences of the Genome Aggregation Database v2.1.1; nf, not found.

## Data Availability

Not applicable.
